# A numerical procedure for understanding the self-absorption effects in laser induced breakdown spectroscopy

**DOI:** 10.1039/d3ra06226k

**Published:** 2023-10-09

**Authors:** Lekha Mary John, K. K. Anoop

**Affiliations:** a Department of Physics, Cochin University of Science and Technology Kochi-682022 India anoopkk@cusat.ac.in

## Abstract

Optical emission spectroscopic techniques, such as laser-induced breakdown spectroscopy (LIBS), require an optimal state of plasma for accurate quantitative elemental analysis. Three fundamental assumptions must be satisfied in order for analytical results to be accurate: local thermodynamic equilibrium (LTE), optically thin plasma, and stoichiometric ablation. But real-life plasma often fails to satisfy these conditions, especially the optical thinness of plasma, resulting in the reabsorption of emitted radiation called self-absorption. To study the self-absorption effect, we simulated optically thick emission spectrum at typical laser-produced plasma conditions. The simulation of the spectrum involves four stages, including the estimation of the ratio of the number density of various ionisation states in the plasma using the Saha–Boltzmann equation, the peak intensity of a spectral line using the Boltzmann equation, the full-width half maxima of each spectral line using the Stark broadening method, and the generation of full spectra by providing a Lorentzian profile for each spectral line. Then self-absorption is applied to the simulated spectrum. We investigated the dependence of the self-absorption coefficient on the plasma temperature, optical path length, and element concentration in the sample. Self-absorption decreases with increased plasma temperature, whereas it increases with increasing optical path length and analyte concentration. We also investigated the role of self-absorption in quantitative analysis by calibration-free LIBS with and without resonance lines of the binary alloy (Mg 50% & Ca 50%). We observed a drastic reduction in error from 27% to 2% in the composition estimation when excluding resonance lines.

## Abstract 2

Self-absorption (SA) of analytical plasmas is studied by simulating optically thick emission spectra. The study found that SA decreases at higher plasma temperatures but increases with longer optical path lengths and higher analyte concentrations.

## Graphical abstract

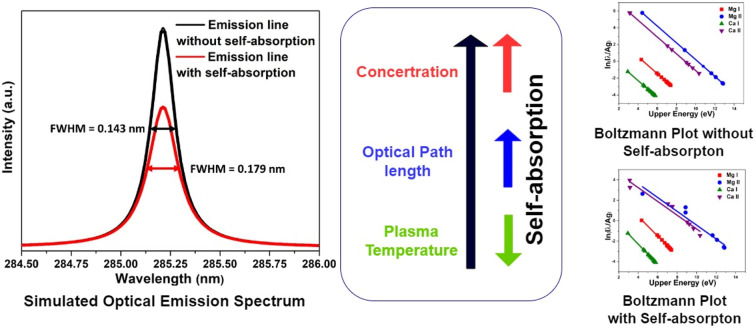

## 1 Introduction

Laser-induced breakdown spectroscopy (LIBS) is an analytical methodology rooted in the principles of atomic emission spectroscopy, which involves the utilization of laser-induced plasma to investigate the elemental composition of a given sample.^1^ The method exhibits notable characteristics such as *in situ* real-time analysis, minimal or no need for sample preparation, and the ability to perform standoff LIBS measurements on materials irrespective of their physical states, which make LIBS relevant among the competitive techniques.^2^ The emission line intensity of each species in the spectrum is related to the species concentration in the sample, and this idea is utilized for quantitative analysis. As a result, any inaccuracy or variation in intensity led to a departure from precise quantitative findings. The precise measurement of intensity is restricted by experimental factors such as the effectiveness of the detection system, the transmission power of collecting optics, *etc.* Apart from these factors, self-absorption, a consequence of optically thick plasma, remains the greatest concern with regard to emission line intensity. Basically, the plasma can only be regarded as optically thin when the emitted light passes through it and exits without evident attenuation or scattering. In optically thick plasma, when light propagates through the plasma to the outside, the emitted radiation is re-absorbed by the same atomic or molecular species in the transmission path.^3^ Thus, the emission intensity measured beyond the source is reduced. Each spectral line exhibits varying degrees of self-absorption, and the reabsorption of a single emission line is influenced by numerous factors. The phenomenon of self-absorption, which leads to a decrease in spectral line intensity, manifests as a nonlinear relationship between peak intensity and concentrations.^4^ As a result, self-absorption must be compensated during the analysis to evaluate concentration accurately.

Self-absorption is a property of all types of natural extended radiation sources. All emission lines undergo self-absorption; however, the effects vary from barely detectable to extremely strong depending on the line.^5^ Besides reducing the line intensity, self-absorption distorts the emission line profile and enlarges the full-width half maxima (FWHM).^3^ Self-absorption will lead to discrepancies in calculating plasma characteristics like the electron number density and temperature. These factors will ultimately result in inaccurate estimations of the elemental composition. Self-absorption depends on the lower-level population, analyte density, radiation source size, ablation efficiency, and plasma volume.^5^ Depending on their abundance in the plasma, lines originating from either neutral species or ionised species of the corresponding element may be prone to reabsorption.^5^ Self-absorption is more prominent for resonance lines (transition end in the ground state) and transitions with significant transition probability. Self-absorption can be quantified using the self-absorption (SA) coefficient, which is the ratio of self-absorbed line intensity to intensity without self-absorption. And its value is between 0 and 1. The line is entirely self-absorbed if it has a value of zero; if it has a value of one, the line is not self-absorbed.

Numerous articles discussed the physical mechanism of self-absorption in LIBS and their corrective methods from theoretical and experimental perspectives.^3–6^ Novel approaches and methods for self-absorption correction or reduction have garnered significant attention in numerous studies in an effort to reduce this undesirable effect.^7–14^ Different works have been reported in the literature exclusively discussing self-absorption correction. Bulajic *et al.* adopted the curve of growth method for self-absorption correction, which combines intricate numerical analysis with physical expressions.^7^ Sun *et al.* employed a comparatively simpler approach based on the internal reference for the self-absorption correction (IRSAC) method.^11^ The degree of self-absorption has been investigated using experimental approaches such as duplicating plasma emission by placing a spherical mirror in front of it.^15^ It is shown that by utilising this simple technique, optically thick plasma conditions can be rapidly determined. Zheng *et al.* used the plasma image as the primary parameter to evaluate self-absorption and proposed “relative self-absorption coefficient” method.^16^

An accurate compositional analysis using LIBS needs a proper understanding of the physical mechanism of self-absorption and the main factors influencing the self-absorption effect.^17^ In this work, we simulated the optical emission spectrum at the typical condition of laser-produced plasma (LPP). Then inserted self-absorption to each spectral line, which varies depending on the line parameters. The impacts of various parameters such as plasma temperature, optical path length and concentration of analyte on self-absorption are then investigated. By this, we try to convey how the hypothesis of an optically thin plasma alters in a real-life situation and emphasize the significance of using self-absorption correction procedures.

## 2 Theoretical background

### 2.1 Simulation of emission spectra at typical conditions of LPP

This section discusses the theory and different stages in the simulation of optical emission spectra.^18^ We require plasma characteristics for simulation, including the fractional composition of the elements in the plasma as well as the plasma temperature (*T*) and electron number density (*N*_e_). Spectroscopic data such as wavelengths, upper and lower-level energies, degeneracy, and transition probability can be retrieved from standard databases like NIST or Kurucz.^19,20^ The Stark width parameter of lines, which is also needed for simulation, may be found in the Stark-B database.^21^ The input for the simulation is the dataset of all available transitions of elements with known spectroscopic data.

The simulation of the optical emission spectrum includes four stages. The first stage involves calculating the concentration of each ionisation stage in the plasma. In the second stage, the Boltzmann distribution law is employed to calculate the intensity of individual emission lines. The third stage determines individual line broadening, and the fourth stage plots the spectrum based on the broadening. The detailed description of different stages in the simulation was explained in our previous work.^18^ In previous work we used Gaussian broadening, which is attributed to the resolving power of the spectrometer. Here, we used stark broadening as the broadening mechanism for the emission lines, and therefore, the line shape function is Lorentzian. A detailed description of the each stages of the simulation is given in the following sections.

#### 2.1.1 Estimation of contribution of each species in the plasma

The optical emission spectrum of the plasma contains emissions originating from distinct ionization states of an element. Under local thermodynamic equilibrium (LTE) conditions, the relative abundances of each ionization state in an atom can be assessed using the Saha-ionization equation. This equation establishes a linkage between the population of a given ionized state and the immediately subsequent higher state of the same elemental species. Consequently, it becomes a valuable tool for estimating the concentration distribution of individual species within the plasma medium. In this study, we specifically examined the neutral, singly ionized, and doubly ionized states of the elemental species. Employing the Saha-ionization equation facilitated estimation of the respective contributions of these ionization states to the plasma composition and is given by,^22^1
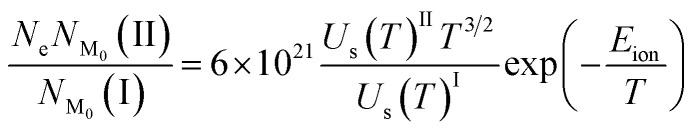
where *N*_e_ is the electron number density, *N*_M_0__(i) and *N*_M_0__(ii) are the species number density in the lower state and subsequent higher state, and *U*_s_(*T*)^I^ and *U*_s_(*T*)^II^ are the partition function of the lower state and subsequent higher state, respectively, at the temperature *T* (in eV), and *E*_ion_ is the ionization energy of the lower state. Using these ratios, we can estimate the fractional contribution of each state of a particular element.

#### 2.1.2 Estimation of peak intensity of individual lines

The Boltzmann equation could be utilized to determine the intensities of the emission lines in the spectrum. The intensity of each line in the spectrum is due to the characteristic transitions of constituent species. The population of each state is distributed according to the Boltzmann distribution law. Therefore, the intensity of the emission line at *λ* wavelength can be expressed in terms of the Boltzmann equation,2
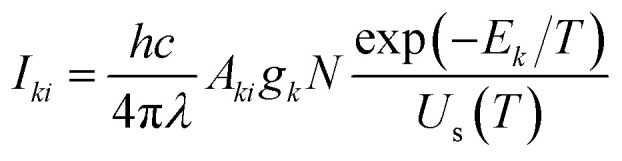
where *I*_*ki*_ is the intensity of the emission line at *λ* wavelength, *N* is the total population of the species, *A*_*ki*_ is transition probability and *g*_*k*_ is the degeneracy of the upper energy level *E*_*k*_. This method pre-computes the intensities of all possible lines at a given temperature and electron density, allowing spectra to be generated over a wide range of wavelengths.

#### 2.1.3 Estimation of full width at half maxima (FWHM) of spectral lines

There are various broadening mechanisms for spectral lines, such as natural broadening, Doppler broadening, and Stark broadening.^1^ Natural broadening in laser-induced breakdown spectroscopy (LIBS) is caused by the finite lifetime of excited states in atoms or ions, leading to an inherent uncertainty in the energy of emitted photons. This uncertainty broadens the emission lines, but it is negligible when compared with other methods.^1^ Doppler broadening of spectral lines is caused by the thermal motion of particles in the plasma. As atoms and ions move at different velocities within the plasma, the Doppler effect leads to a spread in the frequencies or wavelengths of the emitted photons, resulting in broadened spectral lines.^23^ Stark broadening results from the impact of electric fields within the plasma on the energy levels of emitting species. This effect arises due to the interactions between charged particles in the plasma, causing a distortion of the energy levels and broadening the emission lines.^23^ As is well known, Stark broadening is a significant phenomenon in laser-produced plasmas (LPPs), and it results in a Lorentzian line profile. Here we used Stark broadening method to estimate the (FWHM (Δ*λ*_1_/_2_) in nm) of emission lines,^24^3
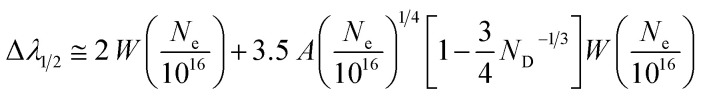
where *N*_e_ is the electron number density, *W* is the electron-impact width parameter, *A* is the ion broadening parameter, and *N*_D_ represents the particle count within the Debye sphere. The value of electron-impact width parameter is taken from Stark-B database.^21^ The electron contribution makes up the first part of the right-hand sides of eqn (1), while the ion correction to the Stark broadening makes up the second. Since electron impact is mainly responsible for Stark broadening of lines, the expression can be simplified to:^25^4
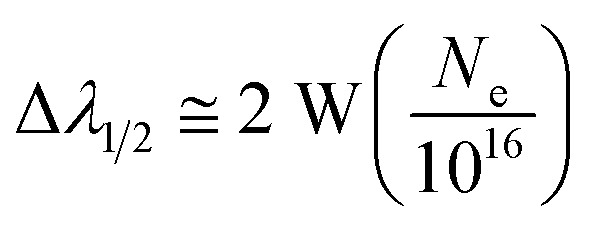


#### 2.1.4 Generating final spectrum

The line profile of laser-induced high-density plasma can be approximately represented by the standard Lorentz shape due to the significant influence of Stark broadening mechanism.^3^ Peak intensity and FWHM are the input parameters for the corresponding wavelengths. The Lorentzian spectral profile is given by,^1^5
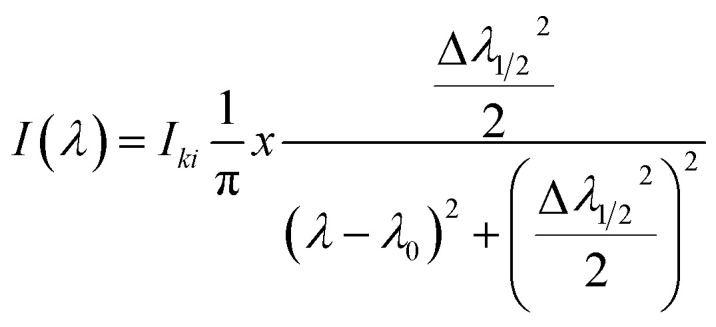


By adding intensity of individual line profile give rise to the full optical emission spectrum of ideal analytical plasma.

### 2.2 Introducing self-absorption to the emission lines

In the real spectrum, emission from plasma suffers self-absorption phenomenon, and its severity varies from line to line. It is essential to evaluate how much does self-absorption influence the analytical line profile. Plasma temperature, optical path length, and analyte concentration in the sample are the primary factors affecting self-absorption. A quantitative parameter must be defined to determine the extend to which self-absorption affects the spectral line. The amount of self-absorption of laser-produced plasma can be determined using the self-absorption coefficient, which is frequently utilized to modify the intensities or widths of spectral lines.^5^ The degree of self-absorption for a specific emission line can be quantified using an assumption of homogenous plasma satisfying LTE.^8^ The self-absorption coefficient, denoted as SA coefficient, is characterized as the ratio between the actual intensity of the emission line when it is under optically thick conditions (*I*(*λ*_0_)) to the intensity value it would possess in a state of optical thinness (*I*_0_(*λ*_0_)). Specifically, its maximum intensity (*i.e.*, for *λ* = *λ*_0_) is lower than that of the optically thin condition, as governed by the following relationship:^3,26,27^6
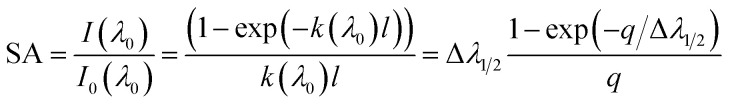
where *k*(*λ*_0_) is the absorption coefficient, and *l* is the optical path length (absorption) (in meters). The *k*(*λ*_0_)*l*, also called as optical depth, determines the self-absorption degree of the line. The Δ*λ*_1/2_ is the FWHM; the formula shows that the SA coefficient and *q* are inversely proportional. The self-absorption effect is high when *q* is large and weak when *q* is small.^8^*q* can be expressed as *q* = 2(*e*^2^/*mc*^2^)*n*_*i*_*fλ*_0_2*l*, where *e* and *m* represent the charge of an electron in units of stat coulombs and the mass of the electron in grams, respectively. And *f* is the oscillator strength of a transition, which is a dimensionless quantity, and *n*_*i*_ represents the number density of the species in the *i*^th^ energy level. When we combine the Boltzmann distribution law and the oscillator strength (the Ladenburg formula), we can express *q* as:^28^7
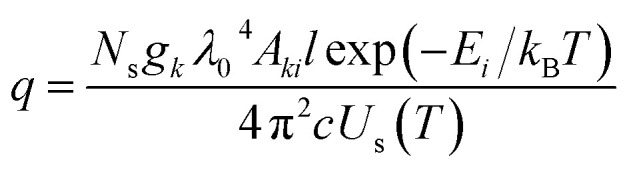
where *λ*_0_ is the emission line wavelength (in meters); *l* is the optical path length (absorption) (in meters); *g*_*k*_ (dimensionless) is the degeneracy (2*J* + 1; *J* represents the total angular momentum quantum number) of the specific state; *N*_s_ is the number density of the species *s* (m^−3^); and *Ε*_*i*_ is the lower-level energy (eV); *T* (K) indicates the plasma temperature; *k*_B_ indicates the Boltzmann constant; *U*_s_(*T*) indicates the partition function. The eqn (6) expresses an inverse relationship between the SA coefficient and *q*. The self-absorption effect is high when *q* is large and weak when *q* is small.^8^ Hou *et al.* investigated how the *q* value changes with the temperature (*T*) of the plasma.^27^ They focused on the Cu I line at 521.82 nm and observed that as the temperature decreases, the *q* value also decreases. However, when they analyzed the Al I line at 396.15 nm, they discovered that the numerical fitting curve of *q versus T* indicated an opposite trend: the *q* value for Al increases as the temperature decreases.^27^ Depending on the abundance of the element in the plasma, self-absorption may be introduced to both neutral and ionised emission lines of the same element. The SA coefficient has a value between 0 and 1. The line is entirely self-absorbed if SA coefficient value is zero and not self-absorbed if it is one. Self-absorption also has an impact on the line width's value.^11,29^ The precise correlation between the observed width (Δ*λ*_1/2_) and the width of the corresponding non-self-absorbed state (*λ*_1/20_) can be mathematically expressed through the utilization of the eqn (6), and the relation given by,^30^8
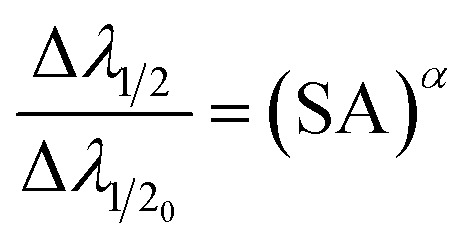
where *α* = −0.54.^30^ Using eqn (6), we can estimate the value of SA coefficient of an emission line, at particular *T*, *N*_s_, and *l* values. And by using eqn (6) and (8), we can estimate the change in line intensity and FWHM to simulate the emission lines accordingly.

## 3 Results and discussion

Self-absorption is not a characteristic of the plasma, as it exhibits variability from line to line within the identical species. The plasma characteristics, such as plasma temperature, species number density, and plasma volume, as well as the spectroscopic characteristics of the line, such as degeneracy, the lower-level energy, and transition probability, all influence the reabsorption of emitted light.^5^ The distribution of population among different energy levels, which subsequently influences the optical thickness of the lines, is strongly influenced by the experimental parameters such as spatio-temporal distribution of the plasma, the laser energy, and the ambient conditions. The influence of these experimental parameters on self-absorption is very complex, and the impact of the experimental parameters must thus be considered separately for different situations and for different emission lines.^5^ Changing the irradiation conditions and the acquisition time may alter the amount of self-absorption in accordance with changes in plasma temperature (*T*), concentration of the species (*C*), and absorption path length (*l*) produced in the LIBS plasmas.^5^

**Fig. 1:**
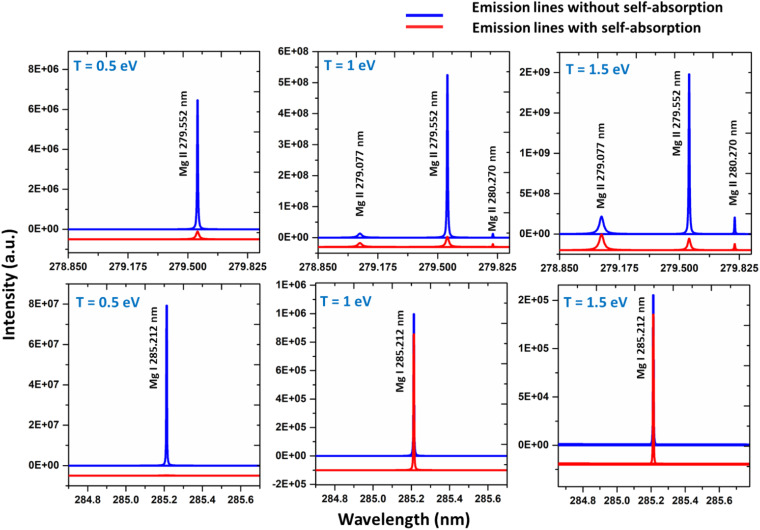
Simulated emission spectrum from optically thick plasma of Magnesium at 1× 10^17^ cm^−3^ electron number density and 0.5 to 1.5 eV plasma temperature (plasma temperature indicated on each graph). For each temperature, emission spectra of neutral and singly ionized species are shown.

**Fig. 2:**
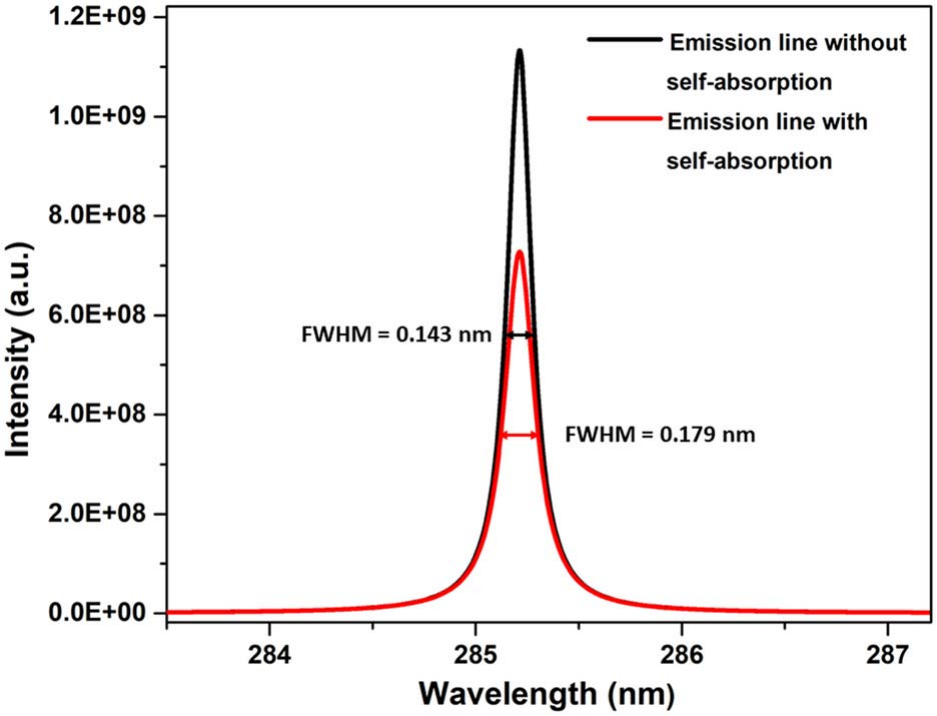
Simulated optical emission line of Mg I at 285.212 nm with and without self-absorption.

We simulated the plasma spectrum of magnesium under both optically thick and thin conditions. These simulations were carried out at three distinct temperatures: 0.5 eV (5802 K), 1 eV (11 604 K), and 1.5 eV (17 407 K). The electron number density was maintained at 1 × 10^17^ cm^−3^, and an optical path length of 1 cm was utilized, reflecting the typical conditions found in laser-produced plasmas (LPPs). In Fig. 1, the impact of self-absorption on the most prominent magnesium spectral lines is demonstrated. Specifically, the lines of Mg I at 285.212 nm and Mg II at 279.077 nm, 279.552 nm, and 280.270 nm are showcased. The spectroscopic parameters for the simulation are taken from NIST spectral database, Kurucz database and the Stark-B database, and the details of the prominent lines are listed in Table 1.^19–21^ A detailed description of the study of the dependence of plasma parameters on self-absorption is given in the next section (3.1). Fig. 2 shows Mg I line 285.212 nm with and without self-absorption simulated at 0.5 eV plasma temperature, 5 × 10^16^ cm^−3^ electron number density, and 0.5 cm optical path length. The purpose of this analysis is to gain insights into the impact of self-absorption on both the intensity and the full-width at half maximum (FWHM) of the spectral line. It is evident that as a line gets more optically thick, its intensity reduces, and its FWHM enlarges. In this work, we investigated how change in the *T*, *C*, and *l* affect the self-absorption of emission lines. Also, we studied the effect of self-absorption in plasma temperature estimation and quantitative analysis.

**Table 1:** Emission lines and corresponding spectroscopic parameters used for simulation

Species	Wavelength (nm)	Stark width parameter (nm)	Transition probability (S^−1^)	Accuracy error of transition probability (%)	Lower energy (eV)	Upper energy (eV)	Degeneracy (lower)	Degeneracy (upper)
Zn I	213.857	0.003	7.14 × 10^8^	3	0	5.795	1	3
Zn I	334.501	0.048	1.70 × 10^8^	10	4.077	7.783	5	7
Zn II	206.200	0.004	3.86 × 10^8^	7	0	6.010	2	2
Zn II	209.992	0.006	5.60 × 10^8^	25	6.119	12.02	4	6
Ca I	239.856	0.114	1.67 × 10^7^	18	0	5.167	1	3
Ca I	318.052	1.670	2.90 × 10^6^	50	1.898	5.796	5	3
Ca I	322.590	3.860	1.60 × 10^7^	50	1.898	5.741	5	7
Ca I	397.371	0.190	1.75 × 10^7^	25	1.898	5.018	5	3
Ca I	422.673	0.012	2.18 × 10^8^	7	0	2.932	1	3
Ca I	443.496	0.126	6.70 × 10^7^	25	1.885	4.680	3	5
Ca I	445.478	0.126	8.70 × 10^7^	25	1.898	4.681	5	7
Ca I	518.885	0.581	4.00 × 10^7^	50	2.932	5.321	3	5
Ca I	585.745	0.790	6.60 × 10^7^	50	2.932	5.048	3	5
Ca II	211.276	0.048	9.70 × 10^7^	25	3.150	9.017	4	6
Ca II	213.230	0.024	2.00 × 10^6^	50	1.692	7.505	4	2
Ca II	317.933	0.045	3.60 × 10^8^	25	3.150	7.049	4	6
Ca II	393.366	0.022	1.47 × 10^8^	25	0	3.150	2	4
Ca II	396.846	0.023	1.44 × 10^8^	25	0	3.123	2	2
Mg I	273.654	2.610	1.25 × 10^7^	25	2.716	7.245	5	7
Mg I	285.166	1.270	2.35 × 10^7^	25	2.716	7.063	5	7
Mg I	285.212	0.007	4.91 × 10^8^	3	0	4.345	1	3
Mg I	294.199	0.237	6.83 × 10^6^	30	2.716	6.929	5	3
Mg I	309.689	0.450	4.96 × 10^7^	18	2.716	6.718	5	7
Mg I	333.667	0.104	1.70 × 10^7^	10	2.716	6.431	5	3
Mg I	383.829	0.151	1.61 × 10^8^	7	2.716	5.945	5	7
Mg I	405.750	4.950	1.02 × 10^7^	18	4.345	7.400	3	5
Mg II	244.959	1.420	2.02 × 10^7^	7	8.863	13.92	4	6
Mg II	266.075	0.881	3.81 × 10^7^	7	8.863	13.52	6	8
Mg II	279.077	0.136	4.01 × 10^8^	3	4.422	8.863	2	4
Mg II	279.552	0.019	2.60 × 10^8^	2	0	4.433	2	4
Mg II	292.863	0.148	1.15 × 10^8^	3	4.422	8.654	2	2

### 3.1 Dependence of plasma parameters on SA coefficient

In order to study the dependence of the plasma parameters on the SA coefficient, we made the assumption that there are three species in the plasma—neutral, first ionised species and second ionization species (notably, the influence of higher-level species on the plasma, specifically for elements Mg, Ca, and Zn, remains negligible, accounting for less than 1% of the total contribution. This observation holds true even at the highest temperature of 1.5 eV considered in our analysis). Each element in the plasma has a constant overall number density. We estimated how the electron number density varies with temperature using the Saha-ionization equation.^31^ We considered confined plasma with optical path length fixed at 1 cm. In this study, we considered Zn, Ca, and Mg plasmas with the total elemental density is approximately 1 × 10^18^ cm^−3^. Next, we varied the plasma temperature from 0.2 eV (2321 K) to 1.5 eV (17 407 K). We employed the Saha-ionization equation (eqn (1)) to determine the ratio of the number density of the first ionized species to that of the neutral species and the ratio of the number density of the second ionized species to that of the first ionized species. As the first ionized and second ionized species contribute electrons to the plasma, the sum of number density of singly ionized species and twice the number density of doubly ionized species equals the electron number density. Furthermore, utilizing the Saha–Boltzmann relation under the assumption of stoichiometric ablation, we derived the number density of neutral species.

**Fig. 3:**
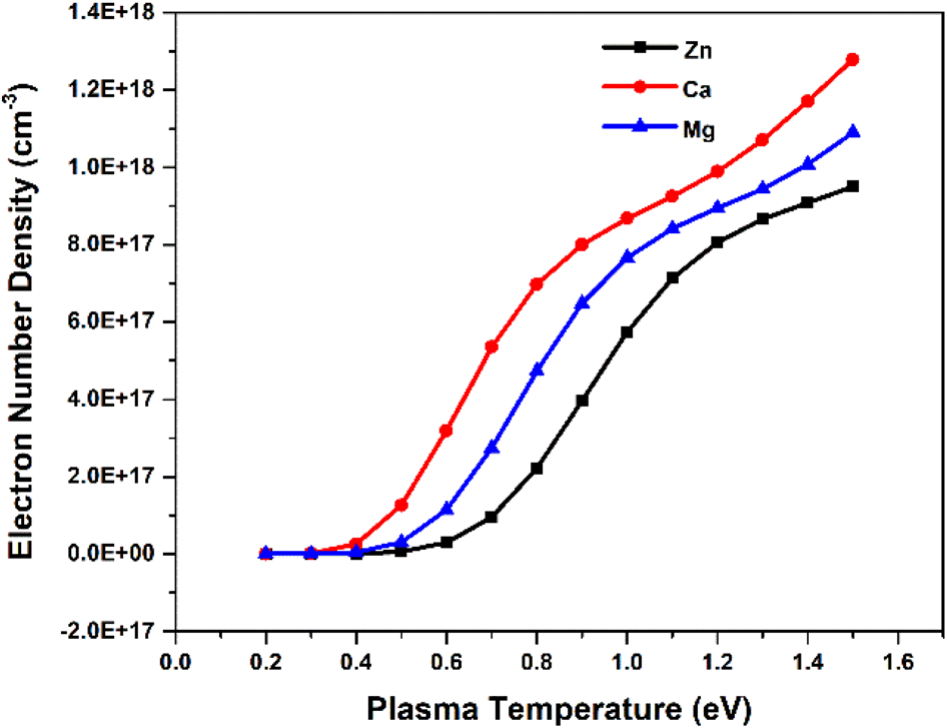
Variation of electron number density of Zn, Ca, and Mg plasmas with temperature.

To investigate the relationship between plasma parameters and the SA coefficient, we considered the plasma of Zn, Ca, and Mg separately, at typical LPP condition. To estimate the electron number density in each step, we raised the temperature by 0.1 eV (between 0.2 and 1.5 eV). The Fig. 3 shows the change in the temperature with electron number density. The change in electron number density with temperature for Zn, Ca, and Mg plasma is solely estimated based on theoretical consideration. Hai *et al.* presented a study on the dependence of plasma parameters on laser fluence in tungsten-copper alloy plasma.^32^ As the laser fluence is raised from 3 J cm^−2^ to 18 J cm^−2^, the plasma temperature exhibits a shift from around 0.7 eV to 1.2 eV, and the corresponding electron number density varies from approximately 5 × 10^15^ cm^−3^ to 5 × 10^16^ cm^−3^.

**Fig. 4:**
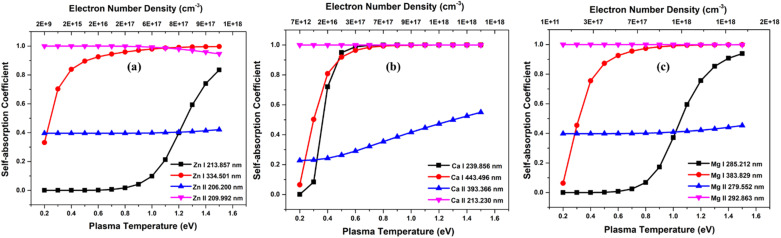
Variation of SA coefficient with electron temperature of (a) zinc, (b) calcium, and (c) magnesium plasmas.

Next, we investigated how plasma parameters affect the SA coefficient. The temperature has been increased for the study from 0.2 eV to 1.5 eV (along with the corresponding electron number density) of Zn, Ca, and Mg plasmas. Fig. 4 depicts the variation of the self-absorption coefficient with plasma temperature for the four lines of each element. Among the lines, we used two neutral lines and two singly ionized lines, and the spectral parameters of each line are tabulated in Table 1. We chose one resonance line among the two neutral and singly ionized lines. As the plasma temperature increases, the self-absorption emission lines reduce for the considered elements. Initially, self-absorption is high, or SA coefficient is low for neutral species of all three elements since the species number density of neutral species is high at low temperatures according to the Saha-ionization relation. As the electron temperature increases, the self-absorption reduces for neutral species. In the case of ionized species, even though the number density of ionized species increases with an increase in temperature, self-absorption shows a slight reduction as the temperature increases. This observation is because the temperature-dependant terms in the eqn (9) reduces *q* value as temperature increases, which dominates the increase in species number density value. That is why we observe a slight decrease in self-absorption as temperature increases, but it is not evident as in neutral lines. In all instances, self-absorption is higher for resonance lines. Hai *et al.* demonstrate that when investigating the Cu I line at 324.75 nm, they observed reduced levels of self-absorption under experimental conditions that create elevated temperature and electron number density.^32^

### 3.2 Variation of SA coefficient with species concentration

**Fig. 5:**
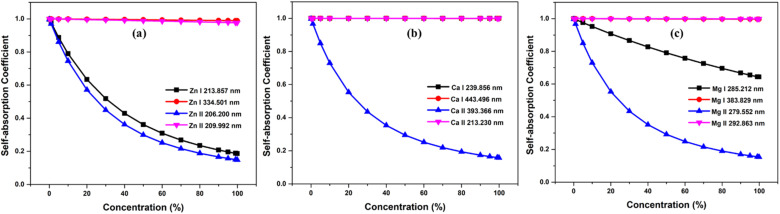
Variation of self-absorption coefficient with concentration of analyte element of (a) zinc, (b) calcium, and (c) magnesium.

To investigate the effect of element concentration on self-absorption, we simulated optical emission spectrum of the alloy plasma composed of the three elements Zn, Ca, and Mg. To compare the self-absorption in each case, the concentration of each element varied from 0.1% to 99.9%. The concentration of the other two elements remains constant, and the total concentration of all three elements adds up to 100%. These simulations were carried out at plasma temperatures of 1 eV, electron number density 1 × 10^17^ cm^−3^, and an optical path length of 1 cm. The Fig. 5 shows the graph of self-absorption coefficient as a function of concentration for three different elements. We used four lines of each of the elements and the spectroscopic data of lines are listed in the Table 1. We choose two neutral and two ionised lines for each element, one of which is the resonance line—a transitional line involving the ground state.

As the concentration of analyte elements in the alloy rises, the SA coefficient reduces, which increases the self-absorption of the emission line. It is evident from the three plots that in all ionisation states, resonance lines show self-absorption to be more prominent. Self-absorption is also extremely low or undetectable for non-resonant lines. In our observation, although self-absorption is present at an analyte concentration as low as 1%, its consequence is more notable at an analyte concentration greater than 15%. For example, in the case of Zn lines in Fig. 5, SA coefficient is greater than 0.85 even for resonance lines at 15% Zn concentration in the sample. Li *et al.* investigate the changes in self-absorption coefficients of spectral lines of Copper (Cu I 324.75 nm) and Chromium (Cr I 427.48 nm) with the variation of elemental concentration in the sample.^33^ For Copper, the concentration in the sample ranges from about 0.02 wt% to 0.35 wt%, leading to a shift in the self-absorption value from roughly 0.9 to 0.25. In the case of chromium, as the concentration in the sample changes from around 0.25 wt% to 2.3 wt%, the self-absorption value changes from approximately 1 to 0.3. In a study by Zhang *et al.*, a significant reduction in the relative SA coefficient values of the lines Mn I at 403.3 nm, Sr I at 460.7 nm, and Li I at 670.8 nm was observed as the concentration increased.^16^ Bulajic *et al.* also observed thickness of plasma for the most prominent lines, such as resonance lines, of elements with concentrations exceeding roughly 0.1%, but it can also occur in less intense lines when the elemental concentrations are higher.^7^ These show that using resonance lines for analytical purposes can result in inaccurate abundance estimation. However, if an element's concentration in the sample is low and there are no other lines with a good signal-to-noise ratio, we can still take resonance lines into consideration for analytical studies, but we must consider self-absorption correction methodologies for the accurate analytical results.

### 3.3 Variation of SA coefficient with optical path length

**Fig. 6:**
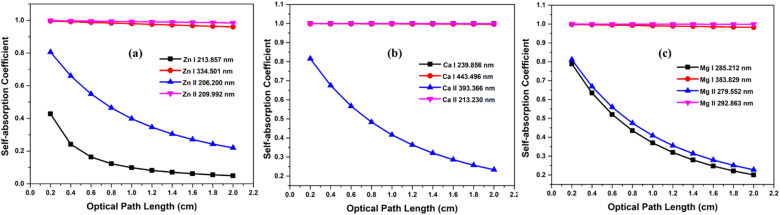
Variation of self-absorption coefficient with optical path length of (a) zinc, (b) calcium, and (c) magnesium plasmas.

The optical path length is the distance through the plasma that the emitted photon needs to travel before escaping the plasma. As is well known, reabsorption is very likely if the photon travels more distance through plasma. We investigated how the SA coefficient varies with optical path length. We considered the neutral and first ionized lines of Zn, Mg, and Ca for the study. The simulation were carried out at plasma temperatures of 1 eV, electron number density 1 × 10^17^ cm^−3^. We changed the optical path length in steps of 0.2 cm, ranging from 0.2 to 2 cm, and found that the self-absorption is very high at larger optical path length, especially for resonance lines. Self-absorption significantly decreases for optically thin plasma, and among non-resonance lines, the variation of the SA coefficient with optical path length is minimal. The Fig. 6 illustrates the variation in optical path length with the SA coefficient. Even at an optical path length of 0.2 cm, the self-absorption effect of resonance lines remains substantial, while the impact of optical path length on self-absorption is minimal for non-resonant lines. Qasim *et al.* investigated the relationship between the SA coefficient value and optical thickness, and they observed that as the optical thickness decreases, the SA coefficient value approaches unity.^34^ Alfarraj *et al.* evaluated the dependence of the optical depths and SA coefficient value of the strontium and aluminum emission lines in the strontium nitrate and aluminum oxide sample spectra.^35^ Their investigation revealed a reduction in the SA coefficient alongside elevated optical depths in situations involving increased analyte concentrations. The level of self-absorption varies based on the regions of plasma the photons pass through, and the degree of self-absorption can differ depending on the angles at which the light is captured.^5,36^ Yi *et al.* found that selecting different positions through spatially resolved laser-induced breakdown spectroscopy can effectively minimize the self-absorption effect of the major elements.^37^

### 3.4 Effect of self-absorption on Boltzmann plot and abundance estimation

Plasma temperature and compositional analysis of laser-produced plasma can be estimated spectroscopically using physical expressions. These physical expressions are based on three fundamental assumptions; plasma is in LTE, optically thin plasma, and stoichiometric ablation. However, it is not always possible to generate optically thin plasma. With the use of calibration-free LIBS, we investigated the impact of deviation of optically thin plasma consideration in plasma temperature and compositional evaluation. Plasma temperature is estimated using the Boltzmann plot method. The following is the Boltzmann equation for the intensity of emission lines:^22^9
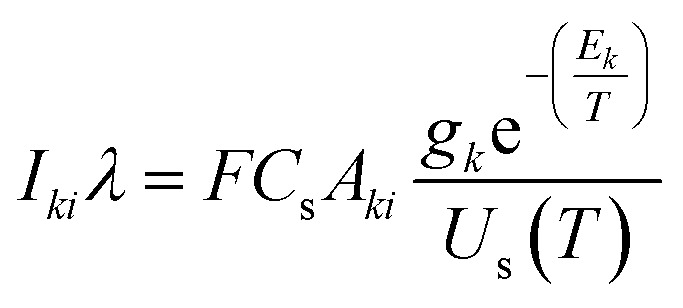
where *C*_s_ representing the concentration of species *s*, *A*_*ki*_ denoting the transition probability between level *k* and *i*, *g*_*k*_ representing the degeneracy of upper state, *E*_*k*_ denoting the upper energy, *U*_s_(*T*) representing the partition function at temperature *T*, and the *F* coefficient depending on the efficiency of the detection system, plasma density and volume. The expression mentioned above (eqn (9)), when converted to a linearized form, is given by,^22^10
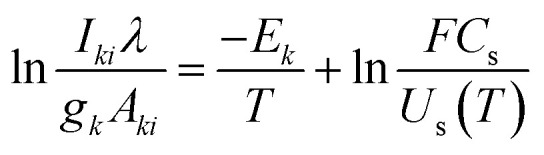


The temperature can be determined by examining the slope of the Boltzmann plot, of 
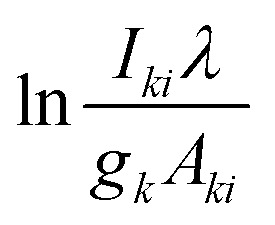
 as a function of *E*_*k*_. For optically thin plasmas in LTE, the lines corresponding to different ionization states must exhibit parallel behaviour. Then the points will be linearly aligned, and the *R*^2^ value of linear regression will be near to one.

**Fig. 7:**
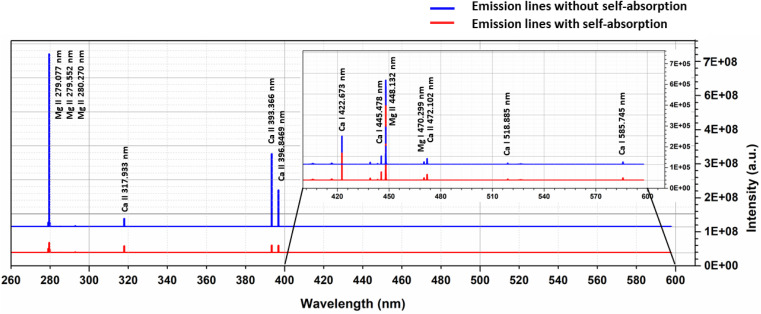
Optical emission spectrum of Mg–Ca alloy (50% Mg & 50% Ca) simulated at 1 eV plasma temperature and 1 × 10^17^ cm^−3^ electron number density. Blue spectrum represents the OES without considering self-absorption effects and red spectrum with self-absorption.

To examine the effects of self-absorption on the Boltzmann plot, we simulated the optical emission spectra of the Ca–Mg alloy (50–50%) at 1 eV plasma temperature and 1 × 10^17^ cm^−3^ electron number density, and the Fig. 7 shows the spectrum. At a plasma temperature of 1 eV, the plasma is more ionized; therefore, the spectrum displays the most prominent lines from singly ionized species. For the element Mg, the spectrum shows strong lines at 279.077 nm, 279.552 nm, 280.270 nm, and 448.132 nm, all belonging to singly ionized states. Among these lines, the ones at 279.552 nm and 280.270 nm are resonance lines and show the highest degree of self-absorption, as observed in Fig. 7. Similarly, the major lines for Ca in the spectrum are 317.933 nm, 393.366 nm, and 396.846 nm, all belonging to singly ionized states. Fig. 7 demonstrates that the intensity of the lines at 393.366 nm and 396.846 nm, which are resonance lines, is mostly reduced due to self-absorption.

**Fig. 8:**
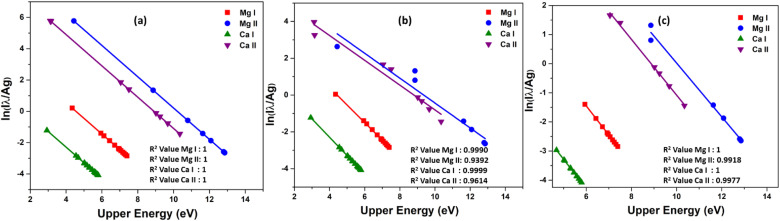
Boltzmann plot of Mg–Ca alloy (50–50%). (a) Boltzmann plot before introducing self-absorption to emission lines, (b) Boltzmann plot of self-absorbed emission lines, and (c) Boltzmann plot of self-absorbed emission lines excluding resonance lines.

The Boltzmann plot is constructed using specific lines from different elements. For Ca I, the lines used are located at 318.052 nm, 322.590 nm, 397.371 nm, 422.673 nm, 445.478 nm, 518.885 nm, and 585.745 nm. The lines from Ca II are found at 211.276 nm, 213.230 nm, 317.933 nm, 373.690 nm, 375.838 nm, 393.366 nm, 396.846 nm, and 472.102 nm. As for Mg I, the lines used are 273.654 nm, 285.166 nm, 285.212 nm, 294.199 nm, 309.689 nm, 333.667 nm, 383.829 nm, and 405.750 nm. Finally, the lines from Mg II are at 244.959 nm, 266.075 nm, 279.077 nm, 279.552 nm, 280.270 nm, 292.863 nm, and 293.650 nm. Table 1 presents the spectroscopic data for these lines. Then, as shown in the Fig. 8, the Boltzmann plot is plotted both before and after the introduction of self-absorption. The figure also includes the *R*^2^ value for the linear fit of each species in the plot. Fig. 8(a) shows the Boltzmann plot without self-absorption, and the *R*^2^ value before introducing self-absorption is almost equal to one for all species, and the temperature evaluated from the slope is also 1 eV for all species. With self-absorption the temperature calculated using Boltzmann plot has significantly diverged from the simulated temperature (Ca I – 1.00 eV, Ca II – 1.48 eV, Mg I – 1.04 eV, and Mg II – 1.48 eV), and the *R*^2^ value of the linear regression is also less than one as shown in Fig. 8(b). For low upper levels energies, the self-absorption effect reduces emission intensity below their theoretical value.^11^ Therefore, the estimated plasma temperatures are higher than the actual plasma temperature.

The scattered points in the Boltzmann plot are mainly due to the resonance lines in each species.^4^ As a result, we do not incorporate resonance lines when plotting the Boltzmann plot; this is depicted in the Fig. 8(c). Then the lines are more linear, and the *R*^2^ value is high. Temperature was estimated from the slope of different species in the plasma is near to simulated temperature (Ca I – 1.00 eV, Ca II – 1.07 eV, Mg I – 1.00 eV, and Mg II – 1.08 eV). The elemental composition is then determined using calibration-free LIBS (CF-LIBS).^22^ We estimated the abundance of neutral and first ionized species in the plasma using the *y*-intercept of the Boltzmann equation (eqn (10)). And the abundance of second ionized species is estimated using Saha-ionization equation (eqn (1)). A small change in the slope of the Boltzmann plot can affect the *y*-intercept and the species abundance estimation.^38^ We determined the concentration of various elements in the plasma, and the results are summarized in Table 2 (placed in the last section of manuscript). The results clearly show that the quantitative results deviate from the standard concentration if we include self-absorbed lines for the quantitative analysis, especially resonance lines. Even though there is a drastic reduction in error in composition estimation by (from 27% to 2%) excluding resonance lines, still, the self-absorption effect can be observed in concentration evaluation, even when conducting quantitative analysis on binary alloys.

**Table 2:** CF-LIBS quantitative result of Mg–Ca alloy with self-absorbed emission lines

Elements	Concentration without SA (%)	Concentration with SA (%)	
		With resonance lines	Without resonance lines
Mg	50	36.42	49.33
Ca	50	63.58	50.67

The self-absorption effect frequently has an impact on the CF-LIBS approach, especially for the major elements. CF-LIBS is superior to the conventional calibration method by introducing a more generalized approach by discarding the use of a calibration curve.^22^ Numerous new and improved CF-LIBS methods are presented with noticeably better quantitative results.^27,38–41^ Self-absorption is a problem for CF-LIBS and calibration curves, so lines without it or lines corrected for self-absorption should be used for calculations.^4^ Compared to the calibration curves method, the self-absorption effect has a greater impact on the sensitivity of the CF-LIBS procedure.^7^ Neglecting this nonlinear impact can be severe, particularly for LIBS quantitative procedures that do not use calibration curves.^4^ Our study shows that we should depend on the self-absorption correction methods to get a precise compositional result.^7,10,11,16,28,42–45^ In addition to self-absorption correction methodologies, there are various methods for overcoming the self-absorption effect.^7,10,11^ One important method is reducing the plasma density by the ambient pressure control method, and the self-absorption coefficient can be brought close to one.^3^ The self-absorption impact on spectral lines was effectively removed by conducting the tests in a vacuum chamber under lowered pressure.

### 3.5 Implications of inaccurate transition probabilities on self-absorption values

**Fig. 9:**
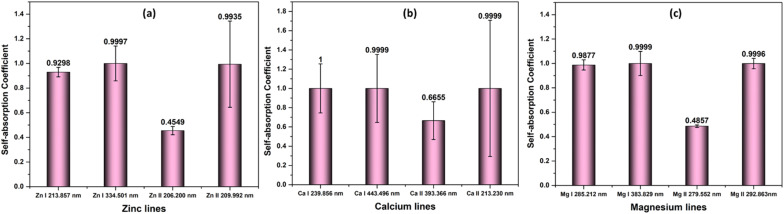
Accuracy error in self-absorption (SA) coefficient of emission lines of (a) zinc, (b) calcium, and (c) magnesium due to error in transition probability.

This research is based on the idea that the values used to calculate SA coefficient are completely accurate, with no errors. However, when it comes to transition probability, there are some uncertainties linked to each line. To explore how these inaccuracies impact SA coefficient values, we utilized two neutral and two first ionized lines from elements Zn, Mg, and Ca (the spectroscopic details of the lines are listed in Table 1). The transition probability of lines suffers varying degrees of accuracy error from 2% to 50%. The details of the accuracy error of each line are taken from NIST spectral database.^19^ The q value in the SA coefficient expression (eqn (6)) is the transition probability-dependent term given in the eqn (7). Therefore, any error in the transition probability will propagate to the SA coefficient value. We simulated self-absorbed lines at 1 eV plasma temperature, 1 × 10^17^ cm^−3^ electron number density, and 1 cm optical path length to study the potential impact of inaccurate transition probabilities on SA coefficient values. In addition to conducting the simulation, we calculated the error in the SA coefficient values using the error propagation equation.^46^Fig. 9 shows accuracy error in SA coefficient of emission lines of zinc, calcium, and magnesium due to error in transition probability. The deviation in the transition probability accuracy for the lines at Zn II 209.992 nm is 25%, and Ca II 213.230 nm is 50%. Consequently, this leads to a notable increase in the error of the SA coefficient values associated with these lines. However, in most considered lines, the error present in the transition probability of the lines is below 20%. As a result, the error linked to the SA coefficient value of these lines is also minimal. It's worth noting that errors in transition probabilities significantly impact compositional analysis. Consequently, we suggest opting for lines with low error rates in transition probability.

Our findings suggest that self-absorption is a global phenomenon that should be considered and eliminated during spectral analysis for the trustworthy evaluation of plasma parameters and elemental composition. Self-absorption is lower for higher plasma temperature and low optical path length. Therefore, experimental plasma confinement to get a high plasma temperature and low optical path length is a suitable method to reduce the effect of self-absorption. Self-absorption is most prominent in the resonance lines and excluding resonance lines while analysis increases the accuracy of results, especially in the case of major constituent elements. We can also consider resonance lines for the minor or trace constituents of samples since the number of lines with a reasonable signal-to-noise ratio is lower for minor components. And self-absorption is a concentration-dependent phenomenon, the minor components suffer less self-absorption than major components. We recommend using lines with high upper energy and low transition probability for better quantitative analysis. And self-absorption correction is always needed for elemental analysis for accurate results.

## 4 Conclusion

We simulated self-absorbed optical emission spectra of different targets under typical laser-produced plasma conditions. We examined how different aspects depend on self-absorption by changing plasma parameters, optical path length, and analyte species concentration. Our study yielded important findings and recommendations, which are outlined below.

High-temperature plasmas with a low electron density effectively minimize self-absorption in emission lines. This can be accomplished by performing experiments at low-pressure conditions and carefully selecting the temporal window for spectral acquisition.

• The optical path length depends on self-absorption, and decreasing the optical path length leads to a less self-absorbed spectrum. We can achieve this by carefully selecting the spatial window for acquiring plasma emission.

• The concentration of analyte species is another significant factor that impacts self-absorption. In particular, it significantly affects the resonance lines of major elements. By excluding resonance lines during quantitative analysis, we can minimize the error in quantitative results.

• CF-LIBS is highly susceptible to self-absorption effects. Skilful experimentation and careful selection of emission lines can enhance the accuracy of quantitative results. However, for greater precision, we recommend employing self-absorption correction methods.

Our study provides valuable insights into the reduction of self-absorption in ideal analytical plasmas. We found that self-absorption is a global phenomenon that must be considered and eliminated during spectral analysis to ensure accurate measurements of plasma parameters and elemental composition. Further experimental and theoretical research is needed to completely eliminate self-absorption effects.

## Author contributions

Lekha Mary John: conceptualization, methodology, software, formal analysis, validation, and writing – original draft. K. K. Anoop: conceptualization, supervision, funding acquisition, validation, writing – review and editing.

## Conflicts of interest

There are no conflicts to declare.

## Supplementary Material
